# Association between diet and the gut microbiome of young captive red-crowned cranes (*Grus japonensis*)

**DOI:** 10.1186/s12917-023-03636-x

**Published:** 2023-06-30

**Authors:** Wei Xu, Nan Xu, Qingzheng Zhang, Keyi Tang, Ying Zhu, Rong Chen, Xinyi Zhao, Wentao Ye, Changhu Lu, Hongyi Liu

**Affiliations:** 1 Co-Innovation Center for Sustainable Forestry in Southern China, Key Laboratory of State Forestry and Grassland Administration on Subtropical Forest Biodiversity Conservation, College of Life Sciences, Nanjing Forestry University, Nanjing, 210037 China; 2 College of Life Sciences, Sichuan Normal University, Chengdu, 610042 China; 3 Institute of Qinghai Tibetan Plateau, Southwest Minzu University, Chengdu, 610041 China; 4 Nanjing Hongshan Forest Zoo, Nanjing, 210028 China

**Keywords:** Red-crowned crane (*Grus japonensis*), Gut microbiome, Age, Diet change, Captive environment

## Abstract

**Background:**

Exploring the association of diet and indoor and outdoor environments on the gut microbiome of red-crowned cranes. We investigated the microbiome profile of the 24 fecal samples collected from nine cranes from day 1 to 35. Differences in the gut microbiome composition were compared across diet and environments.

**Results:**

A total of 2,883 operational taxonomic units (OTUs) were detected, with 438 species-specific OTUs and 106 OTUs common to the gut microbiomes of four groups. The abundance of Dietzia and Clostridium XI increased significantly when the red-crowned cranes were initially fed live mealworms. Skermanella and Deinococcus increased after the red-crowned cranes were fed fruits and vegetables and placed outdoors. Thirty-three level II pathway categories were predicted. Our study revealed the mechanism by which the gut microbiota of red-crowned cranes responds to dietary and environmental changes, laying a foundation for future breeding, nutritional and physiological studies of this species.

**Conclusions:**

The gut microbiome of red-crowned cranes could adapt to changes in diet and environment, but the proportion of live mealworms in captive red-crowned cranes can be appropriately reduced at the initial feeding stage, reducing the negative impact of high-protein and high-fat foods on the gut microbiome and growth and development.

**Supplementary Information:**

The online version contains supplementary material available at 10.1186/s12917-023-03636-x.

## Background

The gut microbiome is composed of many microorganisms that reside and depend on the gut of animals for nutrition, habitat, genetic material, and metabolites. These microorganisms facilitate several host physiological and biochemical functions, including reproduction [1], immunity [2], and digestion [3]. Fecal microbiomes have been used as an index of health condition and phyletic evolution [4]. As the gut microbiome affects the physiological condition of the host, it has been examined in different species from different environments. Further, the gut microbiome can serve as an outstanding indicator of the statuses of rare or agile species at the species and individual levels.

Gut microbes are often used in the study of birds. For example, the gut microbiome has been linked to the productivity of chicken in livestock production [5–7]. A large number of microbes colonize the gastrointestinal tract of chickens, which may play an important role in nutrient degradation [8], development of immune system [9], feed efficiency [10] and so on.

The red-crowned crane (*Grus japonensis*) is listed as a vulnerable bird species by the International Union for Conservation of Nature [11]. Several efforts, including the creation of biosphere reserves and captive breeding programs, have been made to maintain populations by reintroducing captive-bred cranes into the wild [12, 13]. Although the captive population has markedly increased over the last decade [14], infections, malnutrition, and overnutrition can lead to the death of young red-crowned cranes [15–19]. Accordingly, the gut microbiome of juvenile red-crowned cranes could be a helpful index for optimizing the reproductive strategy of cranes to ensure their health and well-being. In the present study, the gut microbial diversity of young red-crowned cranes was analyzed using *16 S rRNA* sequencing and a frequent sampling strategy to reveal the development of the gut microbiome. This strategy aimed to provide a theoretical basis for red-crowned crane breeding, contributing to the growth of the wild populations of this bird species. As a previous study revealed that the gut microbiota of captive red-crowned cranes differs from that of wild red-crowned cranes [13], the results of the present study were further compared with those of Xie et al. to provide a theoretical basis for releasing red-crowned cranes into the wild. Overall, our results imply that changes in the gut microbiome of juvenile red-crowned cranes should be considered important for establishing and improving conservation programs for red-crowned cranes.

## Results

### Bacterial DNA sequencing summary and community characterization

Although DNA was extracted from 30 fecal samples, only 24 of these samples were sequenced as six samples had poor PCR amplification. A total of 3,428,902 raw reads were obtained in both forward and reverse sequencing directions; no reads were lost after assembly. After the initial quality filtering, 3,301,856 sequences were subjected to further analysis. The average (± standard deviation) efficiency of sequencing was 96.32% ± 1.36%, ranging from 91.80 to 97.88%. A total of 2,883 OTUs were detected across all samples using FLASH v1.2.11 according to the Greengenes Database. The number of OTUs in each sample ranged from 43 to 372, with an average of 120 ± 76.

**Fig. 1:**
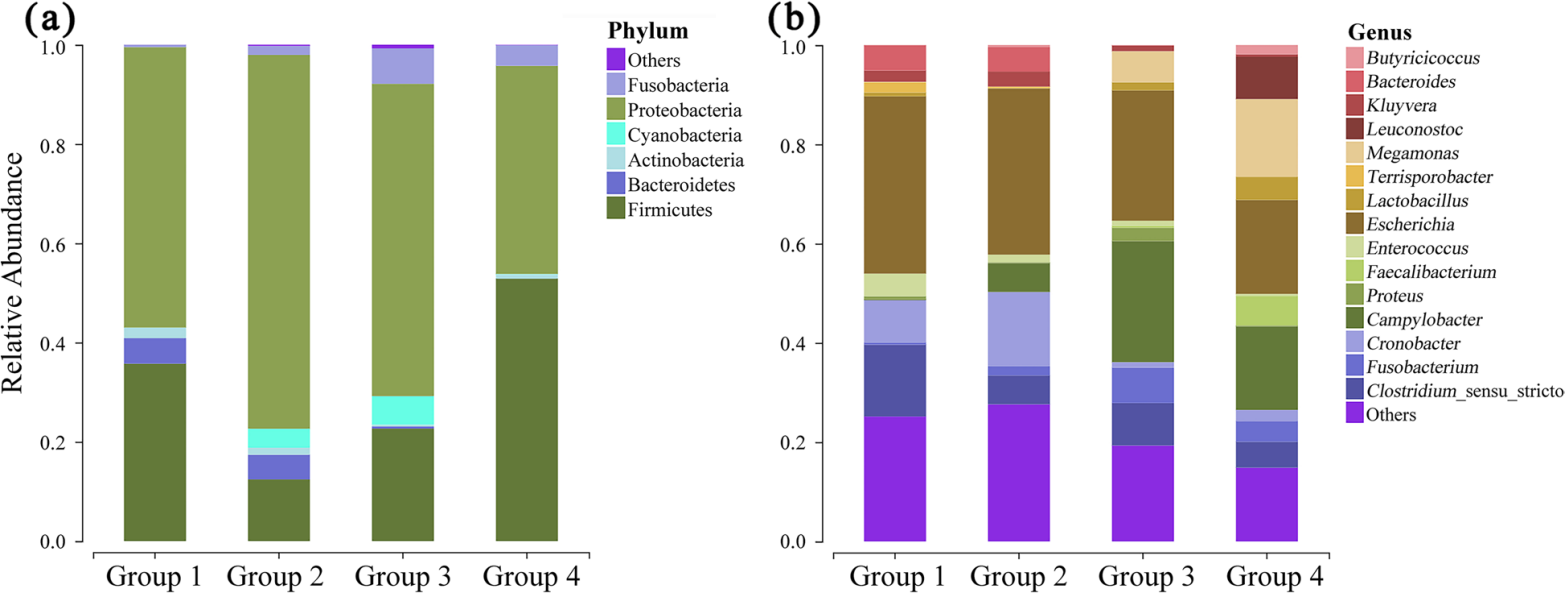
The temporal changes in microbiome relative abundance at the phylum level (Top 6) (**a**); The temporal changes in microbiome relative abundance at the genus level (Top 15) (**b**)

**Fig. 2:**
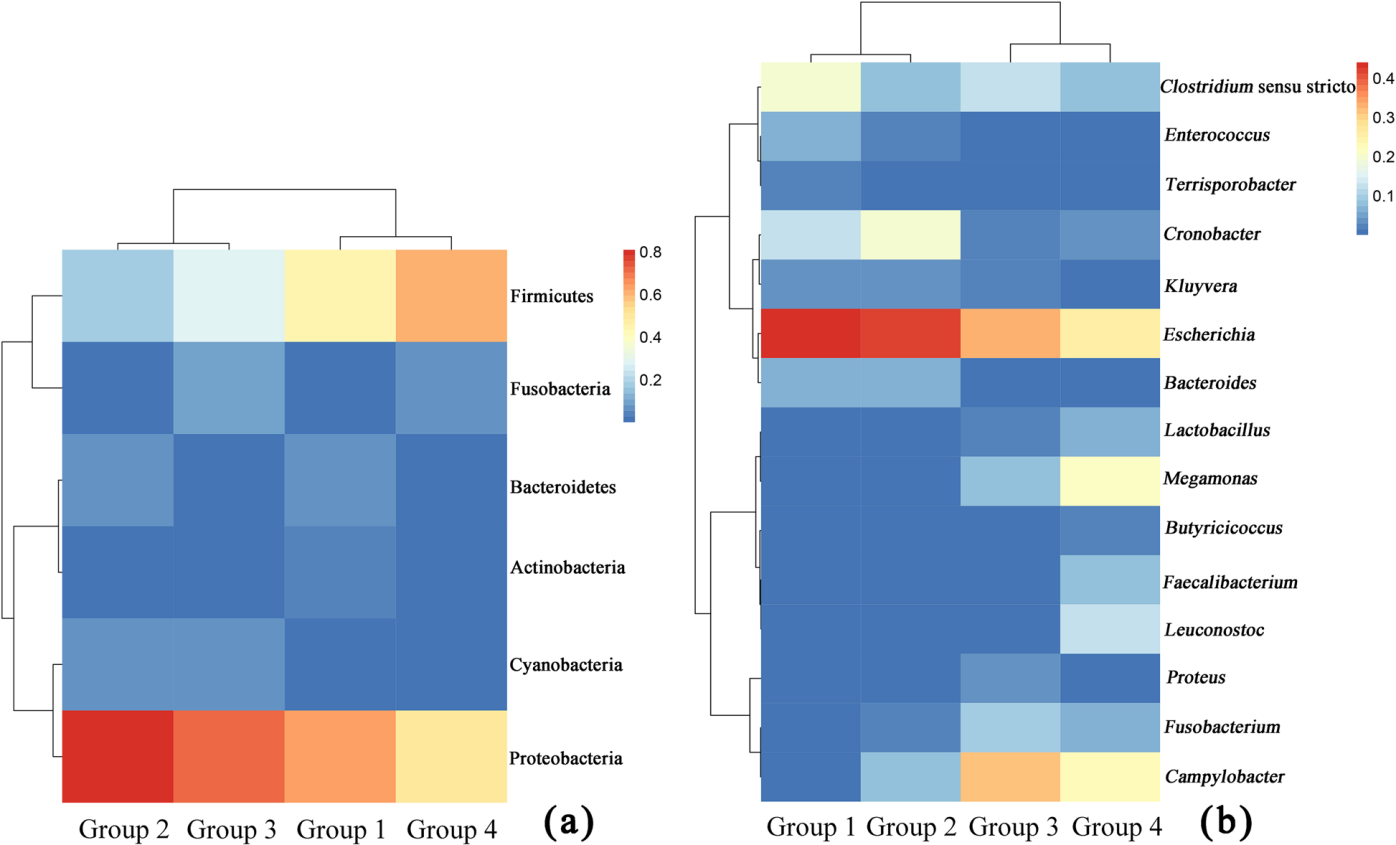
Changes in the relative abundance of the gut microbial species in the four groups. Phylum level (**a**); genus level (**b**)

Overall, six phyla were identified at an abundance = 0.5% (Figs. 1A and 2). At this level, two main differences were found between the groups: Cyanobacteria were not observed in Group 1 and neither Cyanobacteria nor Bacteroidetes were observed in Group 4. The relative abundances of the gut microbiota at the phylum level were similar between Groups 2 and 3 and between Groups 1 and 4. When the OTUs were considered at the genus level, 15 genera had abundance = 0.5% (Figs. 1B and 2). Across all groups, the most abundant genus was *Escherichia* (18.94–35.70%), followed by *Clostridium sensu stricto* (5.22–14.47%). The relative abundances of gut microbiota at the genus level were similar between Groups 1 and 2 and between Groups 3 and 4 (Fig. 2).

### Core microbiota

**Fig. 3:**
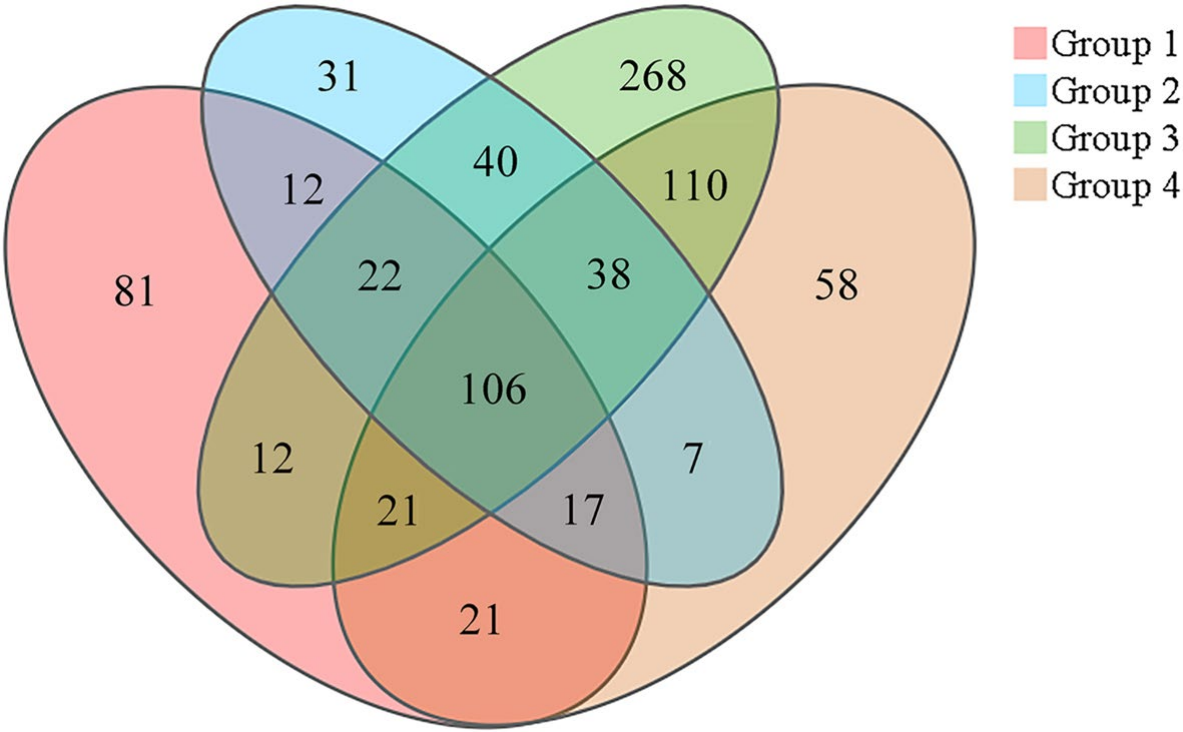
Venn diagram showing the common OTUs in the gut microbiomes of the red-crowned cranes

Both species-specific OTUs (438) and common OTUs (106) were found across the four groups (Fig. 3). In particular, the phyla, Proteobacteria (59.16% ± 13.88%), Firmicutes (30.93% ± 17.47%), and Actinobacteria (1.18% ± 0.75%), were dominant and detected in all groups (Fig. 1A; Table 1). At the genus level, *Escherichia* (28.59% ± 7.59%), *Clostridium sensu stricto* (8.52% ± 4.23%), *Cronobacter* (6.71% ± 6.41%), and *Fusobacterium* (3.40% ± 2.93%) were dominant in the gut microbiome of captive red-crowned cranes (Fig. 1B; Table 1). Nevertheless, their relative abundances varied owing to different factors, such as diet type, environment, and age (Fig. 1).

**Table 1:** Comparison of the predominant bacteria composition of the four groups in the present study to that of a previous study on the gut microbiome of red-crowned cranes

Project	Predominant bacterial phyla (Top 3)	Predominant bacterial genera (Top 5)
This study		
Group 1	Proteobacteria (56.49%), Firmicutes (35.74%), Bacteroidetes (5.13%)	*Escherichia* (35.70%), *Clostridium_sensu_stricto* (14.47%), *Cronobacter* (8.51%), *Bacteroides* (5.10%), *Enterococcus* (4.56%)
Group 2	Proteobacteria (75.29%), Firmicutes (12.43%), Bacteroidetes (4.98%)	*Escherichia* (33.45%), *Cronobacter* (14.99%), *Campylobacter* (5.82%), *Clostridium_sensu_stricto* (5.82%), *Bacteroides* (4.9%)
Group 3	Proteobacteria (62.91%), Firmicutes (22.66%), Fusobacteria (7.14%)	*Escherichia* (26.24%), *Campylobacter* (24.41%), *Clostridium_sensu_stricto* (8.55%), *Fusobacterium* (7.14%), *Megamonas* (6.26%)
Group 4	Firmicutes (52.87%), Proteobacteria (41.93%), Fusobacteria (4.16%)	*Escherichia* (18.94%), *Campylobacter* (16.83%), *Megamonas* (15.64%), *Leuconostoc* (8.51%), *Faecalibacterium* (5.93%)
Xie et al., 2016 [10]	Firmicutes (62.9 ± 4.8%), Proteobacteria (29.9 ± 4.7%), Fusobacteria (9.6 ± 3.0%)	*Enterococcus* (19.1 ± 2.1%), *Bacillus* (12.2 ± 1.5%), *Psychrobacter* (9.3 ± 1.1%), *Lactobacillus* (7.4 ± 1.0%), *Pseudomonas* (5.4 ± 1.7%).

### Gut microbiome development

**Fig. 4:**
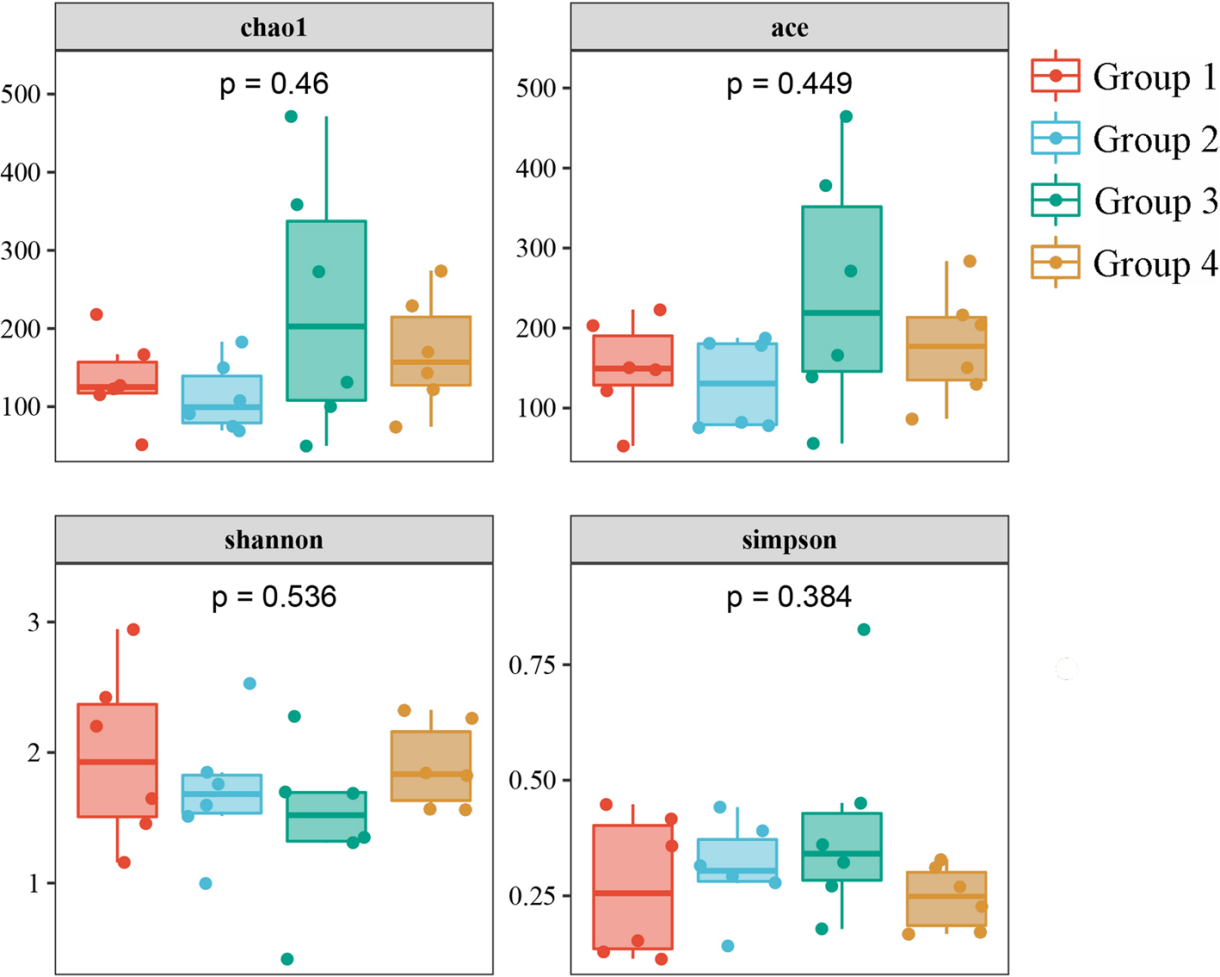
Alpha Diversity (Chao1, Ace, Shannon, and Simpson) between the four groups. The 5 points from bottom to top represent the following: minimum, first quartile, median, third quartile, and maximum. Outliers are denoted by spots

**Fig. 5:**
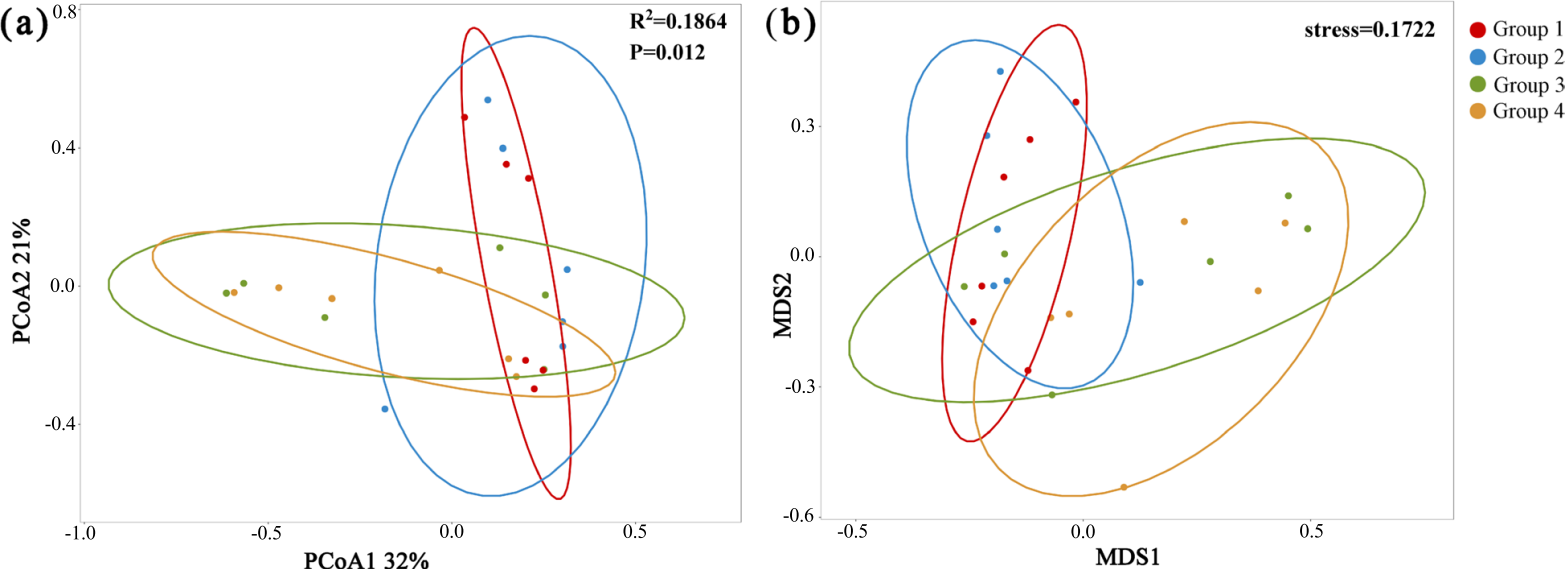
Differences in the gut microbiota of captive red-crowned cranes in the four groups. PCoA results (**a**); NMDS analysis results (**b**)

The composition of the gut microbiome changed over time, as depicted by the differences between the four groups (Fig. 4), specifically with alterations in feeding types and environmental stages. The gut microbiome of Groups 1 and 3 has subtle differences between that of Groups 2 and 4. Although the differences between groups were not significant, the Shannon and Simpson indices displayed opposing trends. However, no particularly significant difference was observed between the Shannon and Simpson indices of the gut microbes for the 24 samples (Supplementary Table 2). This result could be due to the higher sensitivity of the Simpson index to evenness than the Shannon index, and the higher sensitivity of the Shannon index to abundance than the Simpson index. The NMDS (stress = 0.1722) analysis revealed an interweaving among the gut microbiomes of all groups. Further, similarities were noted across the gut microbiomes associated with different diet types (Fig. 5B). However, the PCoA revealed significant differences between each feeding type (*P* = 0.012; Fig. 5A), which might be due to the combined action of feed and environmental (brood box to brood room, to outdoors) changes.

**Fig. 6:**
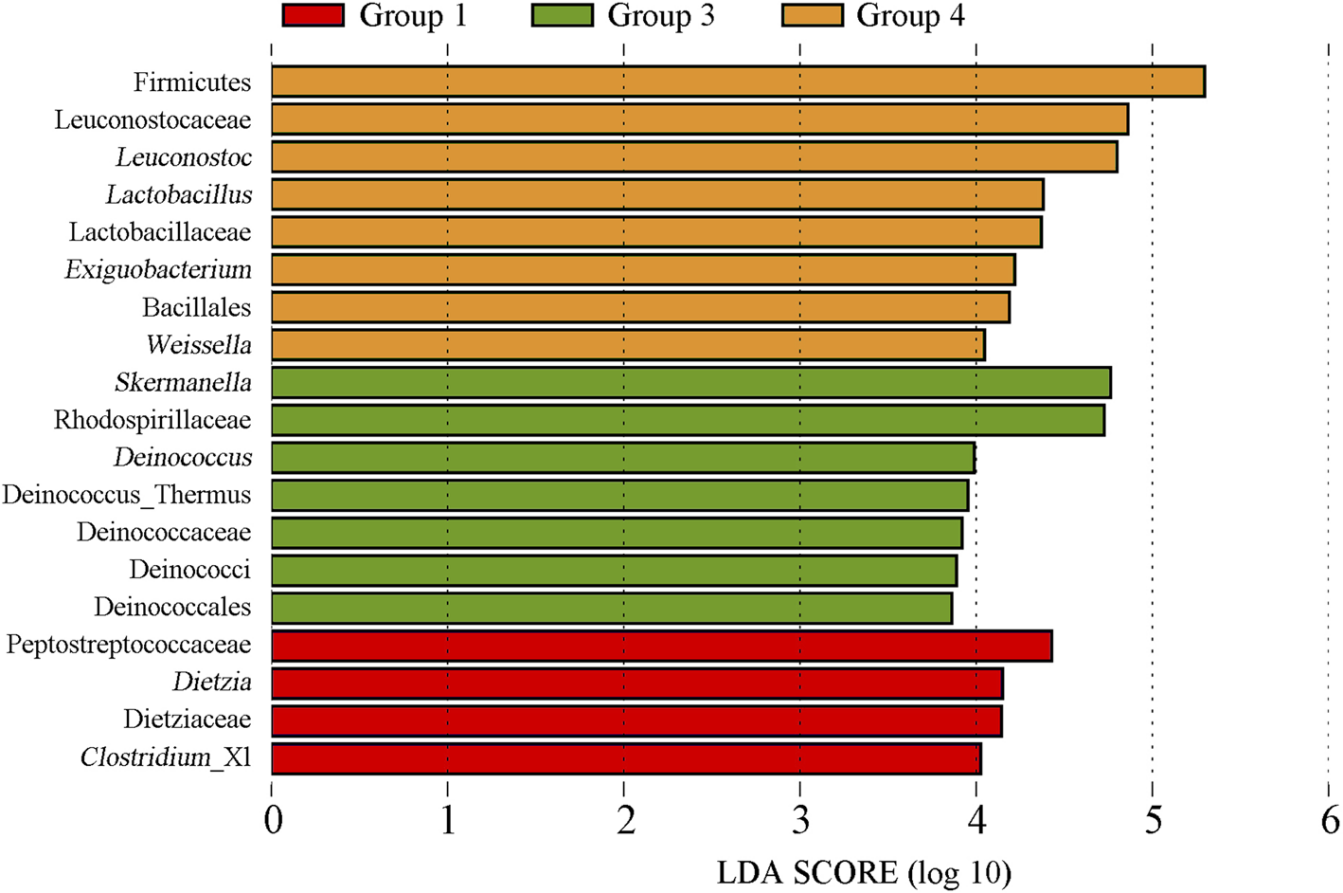
Different colors indicate the microbial taxa that played a significant role in the different groups. It mainly showed the significantly different species with LDA score greater than the preset value, namely Biomaker with statistical difference, the preset value was 2.0. The color of the histogram indicates the length of each group represented by the LDA score

In Group 1, *Dietzia* and *Clostridium* XI were found to be significant taxa based on their LDA score. The gut microbiome of Group 2 was not only devoid of significant taxa, but also showed a lower overall diversity than the other groups. From days 12 to 25, the abundance of *Skermanella* and *Deinococcus* increased significantly. Further, in Group 4, the abundance of *Leuconostoc*, *Lactobacillus*, *Exiguobacterium*, and *Weissella* significantly increased (Fig. 6).

### Molecular pathway analysis

**Fig. 7:**
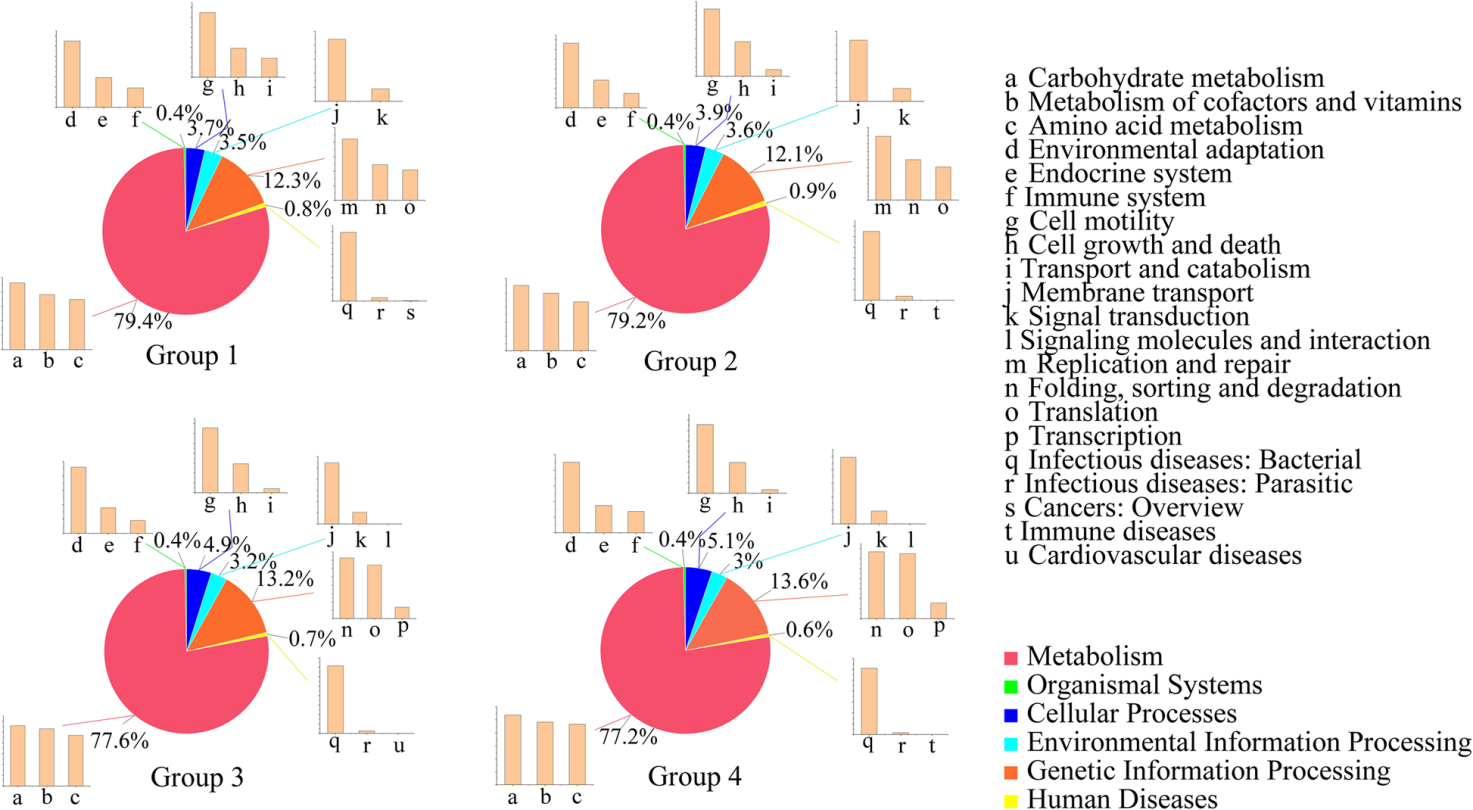
Levels I and II KEGG functional category of the microbiota in the four groups. The pie charts in the middle represent level II pathway categories, and a-u represent the level II pathway categories

The gut microbiota of red-crowned cranes were mainly associated with metabolism (relative abundance, 77.2–79.4%), genetic information processing (12.1–13.6%), and cellular processes (3.7–5.1%) (Fig. 7). The molecular functions were predicted and summarized into KEGG functional pathways and 33 Level II pathway categories. KEGG pathway analysis revealed that the relative abundance of the metabolic pathways decreased with age. Although the number of metabolic pathways did not decrease, that of other pathways more rapidly increased, which also occurred for the cellular process (Supplementary Table 3). The Level II pathways revealed differences among the four groups related to genetic information processing and human diseases.

## Discussion

Among the most abundant gut bacteria found in all four groups, the genus, *Escherichia*, which comprises five species, with *Escherichia coli* as the most important [20], is generally non-pathogenic and found within the normal gut microbiome of humans and animals [21]. *Clostridium sensu stricto* is widely distributed in nature and often exists in the soil, putrefactive substances, and human and animal guts [22]. *Cronobacter* resides in the guts of human and animals and are facultative anaerobic Gram-negative bacteria [23, 24]. Infants and young children are at high risk of developing *Cronobacter* infections, which primarily cause bacteremia, meningitis, and necrotizing enterocolitis [25]. *Fusobacterium* species are normal constituents of the gut microbiome, and are frequently isolated from clinical samples of human and animal origin, especially in cases of pyonecrotic infections [26].

A comprehensive comparison of the richness and composition of the gut microbiome of red-crowned cranes administered different diet types revealed that the gut microbiome of Groups 1 and 3 has subtle differences between that of Groups 2 and 4 (Fig. 4 and Supplementary Table 2). This might be the result of diet and environmental changes [27] but also of the growth and development of the host immune system [28]. The increased diversity of the gut microbiome in Group 1 might be associated with the ingestion of live high-protein mealworms [29]. The diversity of the gut microbiome in Group 2 was lower than that in Group 1, which might be related to the growth and improvement of the autoimmune function or physiological function of red-crowned cranes [28], this finding might also be due to the shorter number of feeding days (only five days). Further, the bird feed, which was added to the diet, contained grains processed at high temperatures, which may have led to feed sterilization, ultimately reducing the number of microbes ingested by the cranes. The highest diversity of the gut microbiome observed in Group 3 might be due to both environmental and diet changes [30]. The composition of the diet in Group 3 and Group 2 markedly varied, and Group 3 was placed in both environmental Stage 2 and Stage 3. Red-crowned cranes were regularly placed outdoors and fed fresh fruits and vegetables after day 11, which could lead to their consumption of a greater number and different types of bacteria from the new diet and environment. This hypothesis is supported by the presence of *Skermanella* and *Deinococcus*, which are widely present in soil, water, and plants, and proliferate in the guts of red-crowned cranes [31–33]. The lower diversity observed in Group 4 might be related to the growth of red-crowned cranes and their improved ability to maintain a stable gut microbiome [28]. *Leuconostoc*, *Lactobacillus*, *Weissella*, and *Exiguobacterium*, which belong to the Firmicutes phylum, were significantly more abundant in this group than the other three groups. Further, the bacterial community composition was similar to that obtained in a previous study on the gut microbiome of adult, wild red-crowned cranes [13]. This finding further supports the greater stability of the structure of the gut microbiome of red-crowned cranes at the later stage of development.

The gut microbiome composition of red-crowned cranes in the present study was compared with that previously obtained for adult, wild red-crowned cranes [13]. As depicted in Table 1, at the phylum level, the microbiome composition of Group 1 did not align with that of adult red-crowned cranes. The abundance of Proteobacteria was high while that of Firmicutes and Fusobacteria was low. However, as age increased, the phylum-level microbiome composition gradually converged with that of adult red-crowned cranes. Notably, some differences were found at the genus level. In fact, the relative abundances of *Campylobacter* and *Clostridium* in the feces of captive cranes were significantly greater than those in the feces of wild cranes [13].

Notably, the administration of live mealworms to newborn red-crowned cranes rapidly increased the number of harmful bacteria in their guts. Living mealworms are rich in bacteria, which leads to an increase in the number of bacteria in the gut microbiome of red-crowned cranes [34]. Of note, harmful bacteria, such as *Dietzia* and *Clostridium* XI, were significantly more abundant in Group 1 than the other groups (Fig. 6). According to previous studies, a high-fat diet can increase the abundance of *Clostridium* in the gut [35, 36]. In addition to causing changes in the gut microbiome, due to the large intake of high protein and high fat at an early age, and insufficient exercise under captive conditions, juvenile red-crowned cranes may become overweight and leg development may be affected [37]. Therefore, the selection and quality control of the starter feed administered to newborn red-crowned cranes must be further investigated and optimized.

## Conclusions

In conclusion, gut microbiome composition and abundance were found to exhibit non-linear changes during the early stages of development of captive red-crowned cranes, with multiple shifts mainly occurring in Proteobacteria and Firmicutes. Based on our findings, diet, environment, and age influence the microbiome structure. Furthermore, changes in the microbiota correlate with diet, environment, and host growth. Herein, the mechanism by which the gut microbiome of red-crowned cranes responds to dietary and environmental changes was revealed, ultimately laying the foundation for future breeding, nutritional, and physiological studies on this species. The results of this study also serve as a basis for improving feed recipes (e.g., reducing live mealworms) and preventing gut colonization by harmful bacteria. Our findings align with those of previous studies on the gut microbiome of rare captive birds and demonstrate the importance of incorporating microbiome research into conservation practices [28, 38, 39].

## Methods

### Breeding environment and diet

**Fig. 8:**
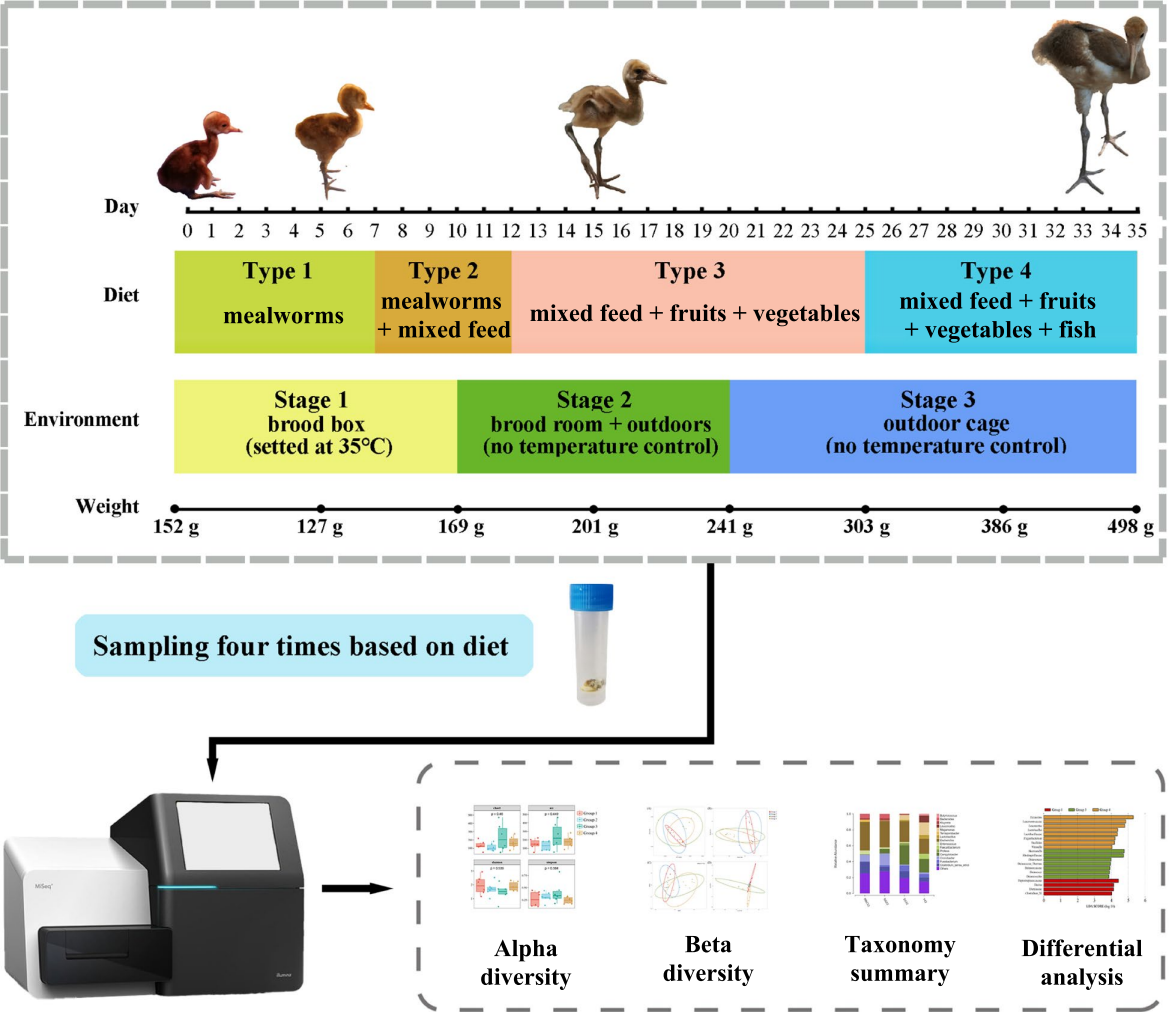
Overview of the study design and sample collection

Fecal samples were collected from nine cranes (six in 2019 and three in 2020) housed at the Nanjing Hongshan Forest Zoo. The cranes were first housed in a brood box at 35 °C; however, with growth, the cranes were transferred to the brood room and then to outdoor enclosures (Fig. 8). Each crane was assigned a number and birthdate based on the information provided by veterinarians and feeders (Supplementary Table 1). Except individual “2019-5,“ who died before Environmental Stage 3, all other individuals experienced three environmental stages. The feed and feeding environment were adjusted according to the temperature and health status of young cranes. The cranes were not fed on the first day of life, but were fed mealworms 1–6 days after birth. Baby bird feed (specially made for cranes) was provided for the subsequent 7 days. Fruits and vegetables were then administered for the next 12 days, and the supply of mealworms was terminated. When cranes were approximately 25 days old, a gradual transition from baby bird feed to adult bird feed was performed. The baby and adult bird feeds had a similar composition (corn, bean pulp, fish meal, bran, bone meal, salt, etc.); however, the proportion of each component in the adult bird food was adjusted to improve digestion and nutrient absorption. Moreover, the baby bird feed was administered in powdered form while the adult bird feed was granular.

### Sample collection

Fecal sample collection was performed at least twice per week. To ensure the quality of the samples, the old feces in the defecation area of young cranes were cleaned in advance. None of the red-crowned cranes was administered antimicrobial drugs during the sampling period. Fecal samples were collected using sterile spoons, placed in tubes, stored in liquid nitrogen, and finally transferred to the laboratory of the Department of Zoology of Nanjing Forestry University for storage at -80 °C. As all baby cranes did not survive, and some uncontrollable factors were encountered in the sampling and sequencing process, only 24 samples were used for the experimental analysis (Table 2). The collected samples were divided into four groups (1–4) according to the feed type and age: Type 1: mealworms; Type 2: mealworms + mixed bird feed; Type 3: mixed bird feed + fruits + vegetables; and Type 4: mixed bird feed + fruits + vegetables + fish (Table 2; Fig. 8). As red-crowned cranes are a threatened species [11], controlled experiments could not be conducted; therefore, no control group was used in this study.

**Table 2:** Information on the fecal samples used in the present study

Sample ID	Individual number	Sampling date	Individual age (days)	Diet type	Group
A19502	2019-5	2019.5.26	2	Type 1	Group 1
A19602	2019-6	2019.6.29	2	Type 1	Group 1
A19402	2019-4	2019.5.26	3	Type 1	Group 1
A20201	2020-2	2020.5.7	4	Type 1	Group 1
A20101	2020-1	2020.5.7	5	Type 1	Group 1
A20301	2020-3	2020.5.12	5	Type 1	Group 1
A19302	2019-3	2019.5.14	5	Type 2	Group 2
A20202	2020-2	2020.5.10	7	Type 2	Group 2
A20302	2020-3	2020.5.14	7	Type 2	Group 2
A19202	2019-2	2019.5.14	8	Type 2	Group 2
A19403	2019-4	2019.5.31	8	Type 2	Group 2
A20102	2020-1	2020.5.10	8	Type 2	Group 2
A20203	2020-2	2020.5.14	11	Type 3	Group 3
A19504	2019-5	2019.6.8	15	Type 3	Group 3
A19404	2019-4	2019.6.8	16	Type 3	Group 3
A19103	2019-1	2019.5.9	17	Type 3	Group 3
A20303	2020-3	2020.5.24	17	Type 3	Group 3
A19604	2019-6	2019.7.17	20	Type 3	Group 3
A19405	2019-4	2019.6.17	25	Type 4	Group 4
A20305	2020-3	2020.6.5	29	Type 4	Group 4
A20205	2020-2	2020.6.2	30	Type 4	Group 4
A19306	2019-3	2019.6.9	31	Type 4	Group 4
A19605	2019-6	2019.7.28	31	Type 4	Group 4
A20105	2020-1	2020.6.5	34	Type 4	Group 4

### Bacterial DNA extraction and library construction

Fecal samples were sent to BGI (Shenzhen, China) for bacterial community DNA extraction using the MagPure Stool DNA KF kit B (Magen Biotechnology Co. Ltd., Guangdong, China), according to the manufacturer’s instructions. DNA was quantified in a Qubit Fluorometer using a Qubit dsDNA BR Assay kit (Invitrogen, Waltham, MA, USA) and its quality was checked on a 1% agarose gel.

The variable V3–V4 region of the bacterial *16 S rRNA* gene was amplified using the degenerate PCR primers, 341 F (5'-ACTCCTACGGGAGGCAGCAG-3') and 806R (5'-GGACTACHVGGGTWTCTAAT-3'). Both forward and reverse primers were tagged with adapters, pads, and linker sequences (Illumina Inc., San Diego, CA, USA). PCR amplification was performed in a 50-µL reaction containing 30 ng of DNA template, fusion PCR primers, and a PCR master mix. The PCR cycling conditions were as follows: 94 °C for 3 min; followed by 30 cycles of 94 °C for 30 s, 56 °C for 45 s, and 72 °C for 45 s; and a final extension at 72 °C for 10 min. The PCR products were purified using AmpureXP beads (Beckman Coulter Inc., Brea, CA, USA) and eluted with elution buffer. The libraries were qualified using the Agilent 2100 Bioanalyzer (Agilent Technologies Inc., Santa Clara, CA, USA). Thereafter, the validated libraries were used for sequencing on the Illumina MiSeq platform at BGI, following the standard pipelines of Illumina; 2 × 300 bp paired-end reads were generated.

### *16S rRNA* sequencing and data processing

Raw reads were filtered to remove adaptors and low-quality and ambiguous bases. Paired-end reads were then added to the tags using Fast Length Adjustment of Short Reads (FLASH, v1.2.11) [40]. The tags were clustered into OTUs with a cutoff value of 97% using UPARSE v7.0.1090, and chimera sequences were detected using the Genomes Online database (GOLD, https://gold.jgi.doe.gov) and UCHIME v4.2.40 [41, 42]. The OTU representative sequences were then taxonomically classified using Ribosomal Database Project (RDP) Classifier v2.2 (http://rdp.cme.msu.edu), with a minimum confidence threshold of 0.6, and aligned on the Greengenes Database v201305 (https://greengenes.secondgenome.com) using QIIME v1.8.0 [43]. USEARCH_global was used to trace all tags to the OTUs to obtain the OTU abundance statistics for each sample [44].

### Bioinformatics analysis

Sample clustering was conducted using QIIME v1.8.0 [43] based on the unweighted pair group method with arithmetic mean (UPGMA). Bar plots for the different classification levels were obtained in R v3.4.1 (https://www.r-project.org). The Venn diagram of the OTUs was plotted using the R package, “VennDiagram” v3.1.1. The alpha diversity at the OTU level was estimated using MOTHUR v1.31.2 [45] and QIIME v1.8.0 [43]. Principal Coordinates Analysis (PCoA) and nonmetric multidimensional scaling (NMDS) based on the Bray-Curtis distance [46] were performed using the R packages, “ape” and “vegan,” respectively. A permutation test was performed using the “adonis” function of R, with a sampling number of 9999. Linear discriminant analysis (LDA) was conducted using linear discriminant analysis effect size (LefSe). Bacterial metagenomes were predicted using the Greengenes Database vgg_13_5, and functional profiles were inferred from the Kyoto Encyclopedia of Genes and Genomes (KEGG) using the phylogenetic investigation of communities by reconstruction of unobserved states (PICRUST2) [47–49].

## Supplementary information

**Additional file 1.**

**Additional file 2.**

**Additional file 3.**

## Data Availability

All of the data used or analyzed during this study are available from the corresponding author on reasonable request. Representative nucleic acid sequences reported in this paper have been submitted to NCBI (https://www.ncbi.nlm.nih.gov/) GenBank database under the accession numbers PRJNA823535.
