# Supplementary material for: Association between diet and the gut microbiome of young captive red-crowned cranes (Grus japonensis)
Source: BMC Vet Res. 2023 Jun 30;19:80. doi: 10.1186/s12917-023-03636-x (PMC10311889; doi:10.1186/s12917-023-03636-x)
Supplement: Supplementary file 1 — Additional file 1. [file 12917_2023_3636_MOESM1_ESM.docx]

**Supplementary Table 1**

| Individual number | Species | Birthdate |
| --- | --- | --- |
| 2019-1 | *Grus japonensis* | 2019.4.24 |
| 2019-2 | *Grus japonensis* | 2019.5.7 |
| 2019-3 | *Grus japonensis* | 2019.5.10 |
| 2019-4 | *Grus japonensis* | 2019.5.24 |
| 2019-5 | *Grus japonensis* | 2019.5.25 |
| 2019-6 | *Grus japonensis* | 2019.6.28 |
| 2020-1 | *Grus japonensis* | 2020.5.7 |
| 2020-2 | *Grus japonensis* | 2020.5.7 |
| 2020-3 | *Grus japonensis* | 2020.5.12 |
