# Supplementary material for: Association between diet and the gut microbiome of young captive red-crowned cranes (Grus japonensis)
Source: BMC Vet Res. 2023 Jun 30;19:80. doi: 10.1186/s12917-023-03636-x (PMC10311889; doi:10.1186/s12917-023-03636-x)
Supplement: Supplementary file 2 — Additional file 2. [file 12917_2023_3636_MOESM2_ESM.docx]

**Supplementary Table 2**

| Sample ID | Group | chao1 | ace | shannon | simpson |
| --- | --- | --- | --- | --- | --- |
| 19502A | Group 1 | 51.2000 | 52.7501 | 1.1574 | 0.4167 |
| 19602A | Group 1 | 167.0000 | 203.5469 | 1.6507 | 0.4478 |
| 19402A | Group 1 | 123.3000 | 122.0392 | 2.4246 | 0.1294 |
| 20201A | Group 1 | 115.3333 | 150.2278 | 1.4593 | 0.3584 |
| 20101A | Group 1 | 127.0000 | 148.4669 | 2.2036 | 0.1526 |
| 20301A | Group 1 | 218.1071 | 222.9456 | 2.9449 | 0.1141 |
| 19302A | Group 2 | 91.2500 | 78.0554 | 1.8478 | 0.2773 |
| 20202A | Group 2 | 107.6000 | 187.7526 | 0.9969 | 0.4418 |
| 20302A | Group 2 | 149.7500 | 180.7778 | 2.5318 | 0.1419 |
| 19202A | Group 2 | 75.1250 | 82.4626 | 1.6004 | 0.3907 |
| 19403A | Group 2 | 183.1429 | 178.9239 | 1.7618 | 0.2928 |
| 20102A | Group 2 | 69.5833 | 75.5963 | 1.5136 | 0.3155 |
| 20203A | Group 3 | 100.1667 | 138.9982 | 0.4207 | 0.8264 |
| 19504A | Group 3 | 131.9091 | 166.1315 | 1.3517 | 0.4508 |
| 19404A | Group 3 | 471.4912 | 464.6290 | 1.6962 | 0.3217 |
| 19103A | Group 3 | 358.7742 | 378.3566 | 1.3099 | 0.3606 |
| 20303A | Group 3 | 273.2500 | 271.6211 | 2.2791 | 0.1783 |
| 19604A | Group 3 | 49.8750 | 55.7172 | 1.6873 | 0.2710 |
| 19405A | Group 4 | 74.3750 | 86.6269 | 1.5645 | 0.3111 |
| 20305A | Group 4 | 170.2308 | 203.8806 | 1.5692 | 0.3290 |
| 20205A | Group 4 | 122.3333 | 130.1334 | 1.8223 | 0.2696 |
| 19306A | Group 4 | 143.5000 | 150.2601 | 1.8448 | 0.2278 |
| 19605A | Group 4 | 229.3333 | 216.2518 | 2.2637 | 0.1677 |
| 20105A | Group 4 | 274.0286 | 283.6311 | 2.3260 | 0.1721 |
