# Supplementary material for: Association between diet and the gut microbiome of young captive red-crowned cranes (Grus japonensis)
Source: BMC Vet Res. 2023 Jun 30;19:80. doi: 10.1186/s12917-023-03636-x (PMC10311889; doi:10.1186/s12917-023-03636-x)
Supplement: Supplementary file 3 — Additional file 3. [file 12917_2023_3636_MOESM3_ESM.docx]

**Supplementary Table 3**

| Items | Group 1 | Group 2 | Group 3 | Group 4 |
| --- | --- | --- | --- | --- |
| Cellular Processes | 55899.12 | 58092.85 | 98967.32 | 98563.1 |
| Environmental Information Processing | 53093.48 | 52916.03 | 64983.8 | 58618.37 |
| Genetic Information Processing | 186230.8 | 179400.2 | 267614.8 | 263735.6 |
| Human Diseases | 12435.21 | 13759.17 | 14806.93 | 12547.24 |
| Metabolism | 1204787 | 1177693 | 1574143 | 1493804 |
| Organismal Systems | 5840.67 | 5697.983 | 7292.594 | 7628.403 |
